# Functional and Morphological Correlations before and after Video-Documented 23-Gauge Pars Plana Vitrectomy with Membrane and ILM Peeling in Patients with Macular Pucker

**DOI:** 10.1155/2015/297239

**Published:** 2015-09-03

**Authors:** Wolfgang J. Mayer, Clara Fazekas, Ricarda Schumann, Armin Wolf, Denise Compera, Anselm Kampik, Christos Haritoglou

**Affiliations:** ^1^Department of Ophthalmology, Ludwig Maximilian University of Munich, 80336 Munich, Germany; ^2^Herzog Carl Theodor Eye Hospital, 80335 Munich, Germany

## Abstract

*Purpose*. To assess functional and morphological alterations following video-documented surgery for epiretinal membranes. *Methods*. Forty-two patients underwent video-documented 23-gauge vitrectomy with peeling of epiretinal (ERM) and inner limiting membrane (ILM). Patient assessment was performed before and 3 and 6 months including best corrected visual acuity (BCVA), slit lamp biomicroscopy, SD-OCT, and central 2° and 18° microperimetry. In addition, all video-documented areas of peeling on the retinal surface were evaluated postoperatively using an additional focal 2° microperimetry. Retinal sensitivity and BCVA were correlated with morphological changes (EZ and ELM) in the foveal region and in regions of membrane peeling. *Results*. Overall, BCVA increased from 0.6 (±0.2) to 0.2 (±0.2) logMAR after 6 months with an increase in retinal sensitivity (17.9 ± 2.7 dB to 26.8 ± 3.1 dB, *p* < 0.01). We observed a significant correlation between the integrity of the EZ but not of the ELM and the retinal sensitivity, overall and in peeling areas (*p* < 0.05). However, no significant correlation between alterations in the area of peeling and overall retinal sensitivity regarding visual acuity gain could be observed after 6 months (*p* > 0.05). In contrast, overall postoperative retinal sensitivity was significantly decreased in patients with a visual acuity gain lower than 2 lines (*p* < 0.05) correlating with EZ defects seen in OCT. *Conclusions*. Mechanical trauma of epiretinal membrane and ILM peeling due to the use of intraocular forceps may affect the outer retinal structure. Nevertheless, these changes seem to have no significant impact on postoperative functional outcome.

## 1. Introduction

Epiretinal membrane (ERM) formation reflects a number of pathological changes occurring in vitreoretinal junctions. Retinal glial cells, fibrous astrocytes, and Müller cells proliferate and migrate from neurosensory retina, through surface and breaks of the internal limiting membrane (ILM). In most cases, the disease is idiopathic but it can also be seen in eyes following retinal surgery, like vitrectomy or extracapsular lens extraction, in uveitic eyes or following vascular retinal diseases [1–4]. The epiretinal membrane itself is defined as a fine, semitranslucent, nonvascular, fibrocellular membrane on the inner retinal surface along the ILM [1, 2, 5]. Affected patients may present with variable degrees of decrease in visual acuity (VA) and disturbing metamorphopsia or micropsia.

Pars plana vitrectomy with membrane peeling is the current standard treatment for surgical removal of ERM, with reported rates of visual improvement ranging between 67% and 82%. In addition, removing the ILM has been suggested as a measure to prevent cellular reproliferation. Furthermore, a number of recent reports are dealing with an interesting correlation of macular function and morphology using SD-OCT and microperimetry. Disruptions of the photoreceptor inner and outer segment band seem to be a potential predictor for poor visual recovery in eyes having undergone macular surgery and some patients also seem to have paracentral microscotomas after membrane and ILM peeling. These findings were postulated to range between 16.6% and 56.2% [6–10].

However, the induction of a potential mechanical trauma by using end-gripping forceps in areas of epiretinal membrane and ILM peeling resulting in potential functional or morphological damage has not yet been addressed.

The aim of the present study was to analyze the correlation between morphological changes of the outer retina, such as EZ (ellipsoid zone) and ELM (external limiting membrane), and functional parameters, such as retinal sensitivity and visual acuity in the fovea and in the area of ERM and ILM peeling, whether the manual peeling using forceps during surgery has an influence on postoperative functional outcome or not. In order to be able to identify these specific areas all operations were video-documented.

## 2. Methods

### 2.1. Study Population and Surgical Approach

In this prospective, observational nonrandomized study, 42 eyes of 42 patients who were diagnosed with epiretinal membranes, but no other retinal diseases, were included. In all subjects 23-gauge pars plana vitrectomy with peeling of the ERM and ILM was performed at the Department of Ophthalmology, Ludwig Maximilians University of Munich, Germany, between July and December 2013. Patients were informed about the use of their data for this study prior to surgery. All patients suffered from a decrease in best corrected visual acuity (BCVA) below 20/30 Snellen. Furthermore, all patients suffered from visual symptoms like disturbing metamorphopsia and/or micropsia. Patients with severe refractive medium opacity, proliferative diabetic retinopathy, age related maculopathy, advanced glaucoma, history of uveitis, previous retinal surgery, and intravitreal injections were excluded. Standard 23-gauge pars plana vitrectomy was performed by two highly trained vitreoretinal surgeons (Anselm Kampik and Christos Haritoglou). In all cases heavy brilliant blue solution (Fluoron GmbH, Neu-Ulm, Germany) was applied for visualization of the ILM before or after ERM removal. The tissue was removed using end-gripping forceps.

Patients with relevant lens opacification (LOCS III with grade >3 of nuclear and/or cortical and/or posterior subcapsular opacification) underwent a combined surgery with cataract extraction and intraocular lens implantation. The surgery was documented on video in order to be able to postoperatively identify areas where peeling using end-gripping forceps was applied.

### 2.2. Patient Examination

**Figure 1:**
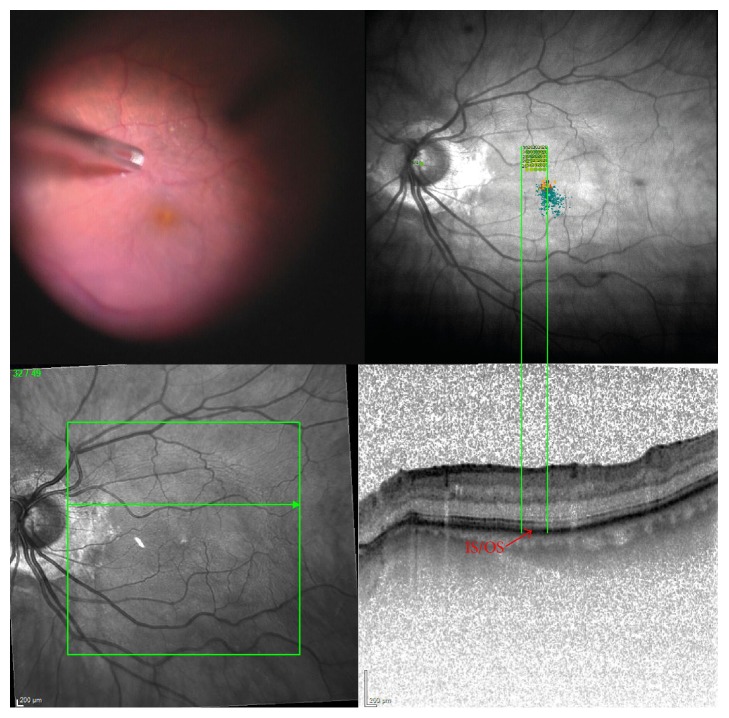
Example for a postoperative evaluation of a peeling area using microperimetry analysis and a matching SD-OCT scan: the integrated photoreceptor junction (IS/OS/ellipsoid zone, bottom right) correlates with a stable retinal sensitivity in this area (green microperimetry field, top right).

All patients were assessed before surgery and 3 and 6 months after the intervention. Preoperatively, a complete medical and ophthalmic history was obtained. A detailed eye examination including measurement of BCVA, intraocular pressure, and slit lamp examination of the anterior segment, with documentation of lens opacities using the Lens Opacities Classification System III, thorough fundus examination by indirect binocular ophthalmoscopy, spectral-domain volume scan OCT (Heidelberg Engineering SD-OCT, Heidelberg, Germany) and central 2-degree and 18-degree microperimetry (MAIA, Ellex Medical Lasers Ltd., Adelaide, Australia) was performed at every visit. In addition, all video-documented areas of manipulation on the retinal surface using forceps were postoperatively evaluated using a focal 2° microperimetry at these areas (Figure 1). Morphological changes in the outer retina such as EZ and ELM were scored using a grading system (grades 0–2) already published in the literature [6, 11] and mean retinal thickness was analyzed with SD-OCT pre- and postoperatively. Briefly, in OCT measurements grade 0 was defined as an intact EZ/ELM junction, as seen by a continuous hyperreflective line, grade 1 showed a focal disruption of the EZ/ELM junction <200 microns in length, and grade 2 was documented as a disruption of the EZ/ELM junction of >200 microns in length. These morphological changes were then correlated with functional results, such as BCVA, expressed as a gain in lines, and mean retinal sensitivity measured by microperimetry.

The macular area was divided into 5 sectors as published previously (modified EDTRS grid) [6] in order to allow a reliable standardized examination and correlation of findings. Sector 1 was defined as the foveal area while the parafoveal area was divided into 4 quadrants labeled sectors 2–5.

Microperimetry was performed with the MAIA machine, which is a near-infrared, line scanning laser ophthalmoscope that incorporates a high frequency eye tracker and an automated macular perimeter to determine threshold sensitivity and fixation characteristics. The automated eye tracker locks onto the entire fundus image and captures fixation changes 25 times per second during testing. The system is using a 4-2-1-staircase strategy with a Goldmann III stimulus. Preoperatively we performed two tests, a 25-stimulus test covering two degrees of the foveal area and a 68-stimulus test covering 18 degrees of the whole macular center field. These two regions were reassessed 3 and 6 months postoperatively. In brief, a fundus image is taken every time a new baseline test is performed with the MAIA machine. A follow-up exam then repeats the baseline expert test by accurately remeasuring the same points while comparing anatomical significant landmarks to the baseline test. In addition, we performed a 25-stimulus test covering two degrees of all video-documented peeling areas to observe any changes in functional sensitivity in these areas during follow-up. Background luminance was set at 4 asb, the stimulus dynamic range was set up to 30 dB, and maximum luminance was 1000 asb.

For the SD-OCT analyses, a volume scan was performed in each observational time step. Five horizontal scans of the fovea and 22 horizontal scans of each parafoveal quadrant with a single scan distance of 11 microns were obtained and separately evaluated in the modified EDTRS grid to cover the areas where peeling of membranes was performed during surgery.

### 2.3. Statistical Analysis

BCVA was measured using a Snellen chart and converted to the logarithm of minimum angle of resolution (logMAR). The Mann-Whitney test was used to compare the statistical distribution of evaluated parameters. Fisher's exact test was used for categorical variable comparison. A change of BCVA of at least 2 Snellen lines was considered statistically significant. The mean retinal sensitivity of the fovea, overall and in areas of peeling, was correlated with mean SD-OCT grading (0–2). All analyses were conducted using SPSS statistics software (SPSS, Inc., Chicago, IL, USA). A *p* value < 0.05 was considered statistically significant. The impact of lens opacities on BCVA was analyzed using Lens Opacities Classification System III.

## 3. Results

The study population consisted of 20 males and 22 females with a mean age of 71 years (range of 45–86 years). In all eyes, epiretinal membranes were successfully removed. In 24 eyes with relevant lens opacification using LOCS III grading we performed combined cataract surgery and vitrectomy; 18 eyes were already pseudophakic. However, impact of lens opacity on BCVA was not significant between the combined surgery group and preoperative pseudophakic group (*p* = 0.12). The video documentation of all surgical procedures revealed that the surgeon made 3 to 8 (mean of 5.3) grasps with the end-gripping forceps at the retinal surface to remove epiretinal membranes and the ILM.

### 3.1. Overall Best Corrected Visual Acuity and Microperimetry Changes

The median preoperative BCVA was 0.6 (±0.2) logMAR. We encountered no postoperative complications in any of the cases, that is, endophthalmitis, retinal detachment, macular edema, or persisting or recurring ERM formation. Overall, 30 out of 42 patients showed an increase of more than two lines 6 months postoperatively. Mean overall macular sensitivity increased from preoperative 17.9 (±2.7) dB to postoperative 24.9 (±3.0) dB after 3 months and to 26.8 (±3.1) dB after 6 months. Mean macular sensitivity increased from preoperative 21.9 (±3.9) dB to postoperative 23.6 (±4.4) dB after 3 months and to 28.4 (±1.6) dB after 6 months in all areas where peeling was initiated (Table 1). Fixation stability increased from 79 (±10.1) percent to postoperative 89.5 (±9.2) percent after 3 months and 97.0 (±11.9) percent after 6 months (Table 1).

**Figure 2:**
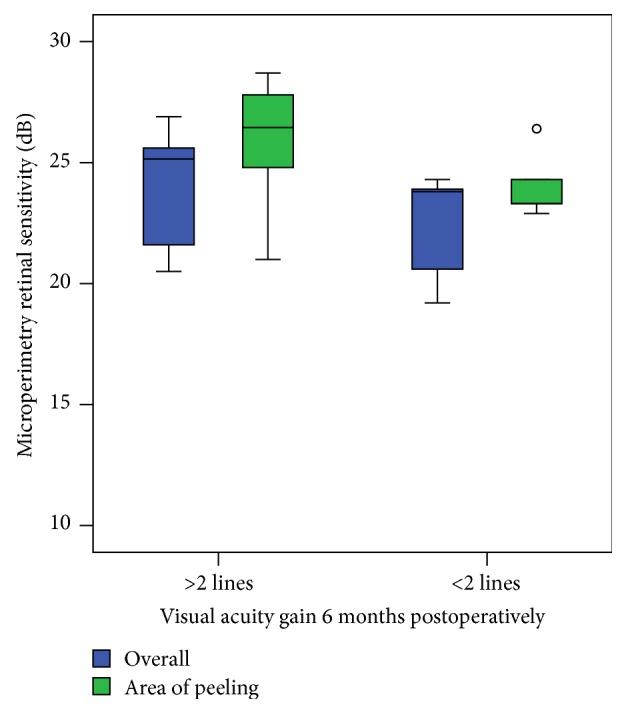
Mean retinal sensitivity in microperimetry measurements overall (18°, blue box) and areas of peeling (2°, green box) based on visual acuity gain 6 months postoperatively. No significant correlation between retinal sensitivity in areas of peeling and overall measurements (*p* = 0.08).

Patients with at least 2 Snellen lines BCVA improvement (*n* = 30) showed an increase of retinal sensitivity in microperimetry in all areas where peeling using end-gripping forceps was initiated during surgery, which was not statistically significant compared to retinal sensitivity in general (*p* = 0.08, Figure 2).

### 3.2. OCT Findings

**Figure 3:**
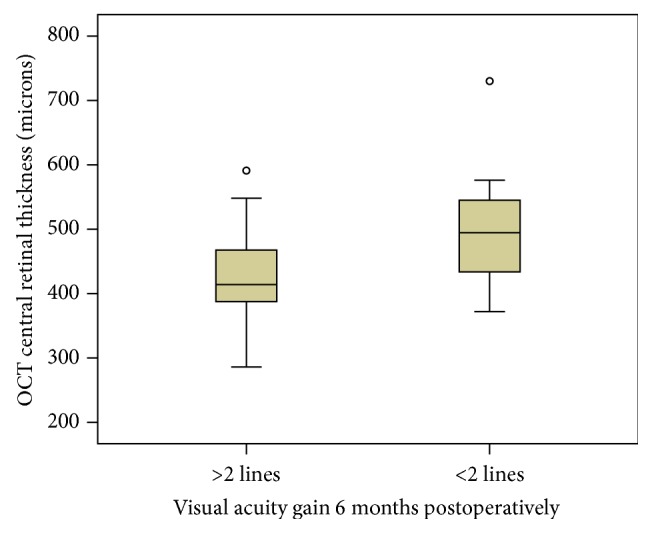
Comparison of preoperative mean central retinal thickness between patients with more than 2 lines (*n* = 30) and patients with less (*n* = 12) than 2 lines of visual acuity gain (*p* < 0.05).

Central retinal thickness decreased from 455.7 (±104.3) microns to postoperative 379.1 (±53.3) microns after 3 months and to 332.3 (±51.1) microns after 6 months. We observed a significant correlation between preoperative central retinal thickness and the increase of BCVA. Patients with an increase of at least 2 Snellen lines (*n* = 30) had a significant thinner preoperative central retinal thickness (*p* < 0.02, Figure 3).

**Figure 4:**
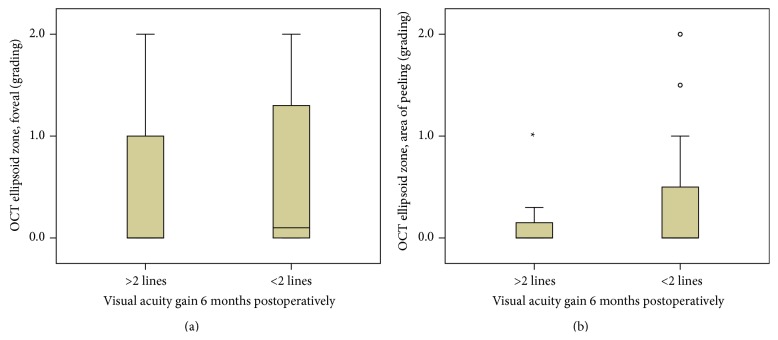
(a) Correlation of preoperative foveal EZ grading and gain of visual acuity 6 months postoperatively. No significant difference between groups (*p* > 0.05). (b) Correlation of preoperative EZ grading in areas of peeling and gain of visual acuity 6 months postoperatively. No significant difference between groups (*p* > 0.05).

**Figure 5:**
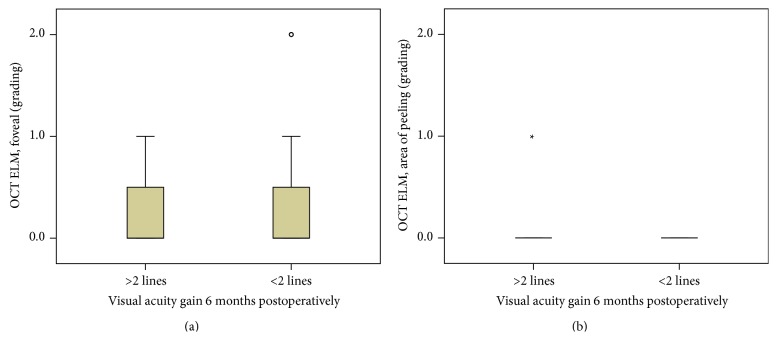
(a) Correlation of preoperative foveal ELM grading and gain of visual acuity 6 months postoperatively. No significant difference between groups (*p* > 0.05). (b) Correlation of preoperative ELM grading in areas of peeling and gain of visual acuity 6 months postoperatively. No significant difference between groups (*p* > 0.05).

Eyes with a preoperative intact ellipsoid zone junction (grade 0) showed a greater improvement in BCVA compared to eyes with an irregular ellipsoid zone junction (grade 1) or disrupted ellipsoid zone junction (grade 2). However, this difference was not statistically significant, for both the foveal region and areas of documented peeling. Quite similar results were obtained for the ELM (Figures 4(a), 4(b), 5(a), and 5(b)).

**Figure 6:**
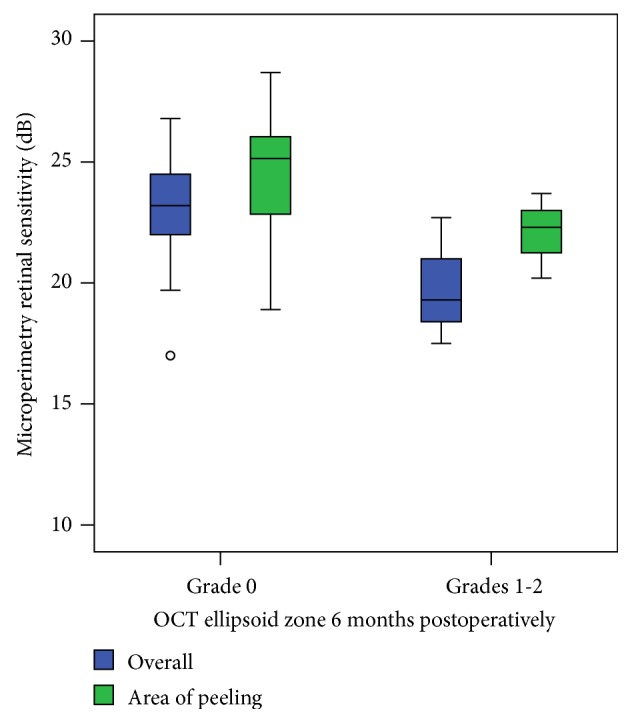
Mean retinal sensitivity based on EZ grading in OCT measurements 6 months postoperatively. A significant difference between intact EZ zone (grade 0) and retinal sensitivity compared to disrupted (grades 1-2) EZ zone in both OCT measurements overall and in areas of peeling could be demonstrated (*p* < 0.05).

We observed a significant correlation between EZ integrity and mean retinal sensitivity postoperatively, both overall and in areas where peeling using end-gripping forceps was initiated (*p* < 0.05, Figure 6). However, alterations in areas of peeling had no significant influence on gain in visual acuity (*p* > 0.05).

Furthermore, investigated OCT measurements in areas of peeling showed no worsening of preexisting outer retinal structure defects or new defects in a preexisting normal EZ and ELM band after surgical intervention.

## 4. Discussion

The current approach of correlating morphological alterations with functional ones in various macular diseases is to compare OCT scans with microperimetry patterns, not only in the foveal but also in the parafoveal area. Due to advances of newer OCT machines (high resolution, eye tracker, and fast scan mode) it is possible to evaluate the vitreomacular interface and outer retinal structures in more detail. Specifically, the EZ, formerly IS/OS, and the ELM have been thought to be of prognostic nature in case of pathological alterations or surgical intervention like pars plana vitrectomy with membrane peeling [6, 8, 9, 11–13].

The formation of ERM is a pathology of the vitreoretinal interface and the cellular proliferation is very likely related to an incomplete posterior vitreous detachment in idiopathic cases but can secondly occur after different retinal diseases or interventions such as retinal breaks, laser or cryotherapy, inflammatory diseases, or retinal detachment. As a first step tractional forces lead to morphological disorganization of the inner retina [14–16] followed by changes in the outer retinal layers as the disease progresses. The extent and localization of outer retinal changes in eyes with ERM formation may be very variable and correlate with the size of the membrane and the focus where tractional forces are most pronounced. These morphological alterations are often associated with a decrease of visual acuity and disturbing metamorphopsia, which are the main indications for surgical intervention [17–20].

However, in some cases the functional result obtained postoperatively is not satisfying for both the surgeon and the patient despite clinically visible anatomic success. The reason for this discrepancy is correlated with alterations of the outer retinal layers, which can be depicted with high resolution imaging OCT [7, 12, 20–22]. Furthermore, our study group recently showed that not only foveal but also parafoveal alterations in photoreceptor junction influence postoperative outcome [6]. In addition to a positive correlation between outer segment restoration and functional results after macular surgery, Itoh and associates observed that a recovery of the foveal cone microstructure may be seen as late as 12 months after anatomically successful surgery and that intact cone outer segment tips after ERM surgery correlate with BCVA [12, 21]. The influence of the used tamponade in the end of ERM surgery (air or balanced salt solution) has also been discussed [23].

However, so far published studies investigating the role of the outer retinal layers and their impact on functional recovery focused mainly on foveal sections obtained during OCT examinations and did not include the area surrounding the fovea [7, 8, 10, 13, 17, 18, 24–27]. The present investigation systematically analyzed both the foveal and the parafoveal region and established a correlation between morphological abnormalities detected in OCT images and retinal function in these specific areas as measured by microperimetry and BCVA after a surgical intervention.

Despite a successful surgical intervention some patients still complain about decreased visual acuity and/or microscotoma postoperatively. To exclude a mechanical injury to the retina by using end-gripping forceps we analyzed in detail the areas of peeling using OCT and microperimetry to compare morphological alterations in the outer retina with functional ones. In our study we could find a significant correlation of decreased retinal sensitivity and EZ or ELM interruptions in OCT measurements both overall and in areas of peeling. However, patients with more than two lines of visual acuity gain showed no significant improvement in retinal sensitivity compared to patients with a visual acuity gain below 2 lines.

Our results indicate that EZ integrity on SD-OCT is a statistically significant predictor of visual acuity in patients with ERM formation, and statistical analysis illustrates that EZ disruption, in contrast to ELM disruption, increases the predictive power of OCT measurements. Furthermore, the present study indicates that the standard surgical approach of ILM and ERM peeling using forceps has no significant negative influence on postoperative retinal sensitivity outcome, even if preexisting outer retinal alterations exist.

A limitation of our study is related to the limited number of patients included and the evaluation of only outer retinal structure alterations.

As we have already shown in our previous study [6], the present work confirms that morphological and functional tests (SD-OCT and microperimetry) in patients with ERM formation should not be focused on the foveal region alone but should also cover the parafoveal area. In addition, the surgical approach using manual forceps for membrane peeling in a highly trained surgeon setting does not influence functional outcome. Therefore, standard 23-gauge vitrectomy with membrane peeling is a safe and efficient approach to treat ERM formation.

## Figures and Tables

**Table 1:** Overall data assessment during 6-month follow-up.

Parameters (*n* = 42)	Baseline	3 months	6 months
BCVA (logMAR)	0.6 (±0.2)	0.3 (±0.3)	0.2 (±0.2)
Central retinal thickness (*µ*m)	455.7 (±104.3)	379.1 (±53.3)	332.3 (±51.1)
Ellipsoid zone, foveal (grading, 0–2)	0.5 (±0.7)	0.3 (±0.5)	0.3 (±0.5)
Ellipsoid zone, area of peeling (grading, 0–2)	0.3 (±0.5)	0.1 (±0.4)	0.1 (±0.3)
Retinal sensitivity, foveal (dB, max. 30)	21.2 (±3.7)	22.5 (±2.6)	23.2 (±2.7)
Retinal sensitivity, overall (dB, max. 30)	17.9 (±2.7)	24.9 (±3.0)	26.8 (±3.1)
Retinal sensitivity, area of peeling (dB, max. 30)	21.9 (±3.9)	23.6 (±4.4)	28.4 (±1.6)
Fixation (%)	79 (±10.1)	89.5 (±9.2)	97.0 (±11.9)
